# P-2148. Infection Following Cytokine Release Syndrome in Patients Receiving Chimeric Antigen Receptor T-Cell Therapy

**DOI:** 10.1093/ofid/ofaf695.2311

**Published:** 2026-01-11

**Authors:** Jack W McHugh, Ella Nadarevic, Douglas Challener, Andre De Menezes Silva Corraes, Yi Lin, Paschalis Vergidis

**Affiliations:** Mayo Clinic, Rochester, MN; Mayo Clinic, Rochester, MN; Mayo Clinic, Rochester, MN; Mayo Clinic, Rochester, MN; Mayo Clinic, Rochester, MN; Mayo Clinic, Rochester, MN

## Abstract

**Background:**

Chimeric antigen receptor T-cell therapy (CAR-T) has transformed care for relapsed or refractory B-cell malignancies. Cytokine-release syndrome (CRS) complicates most infusions, yet the ensuing infection risk is ill-defined.

**Table 1:**
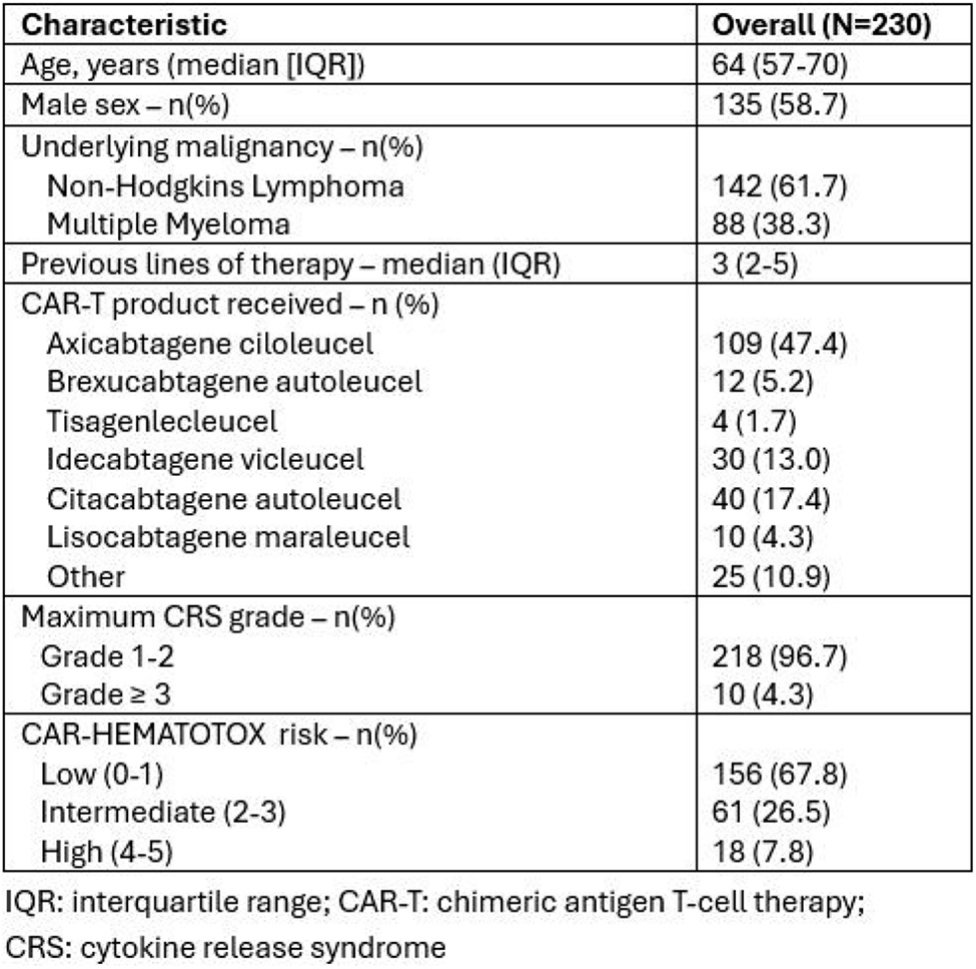
Profile of 230 patients undergoing with CRS following CAR-T therapy

**Table 2:**
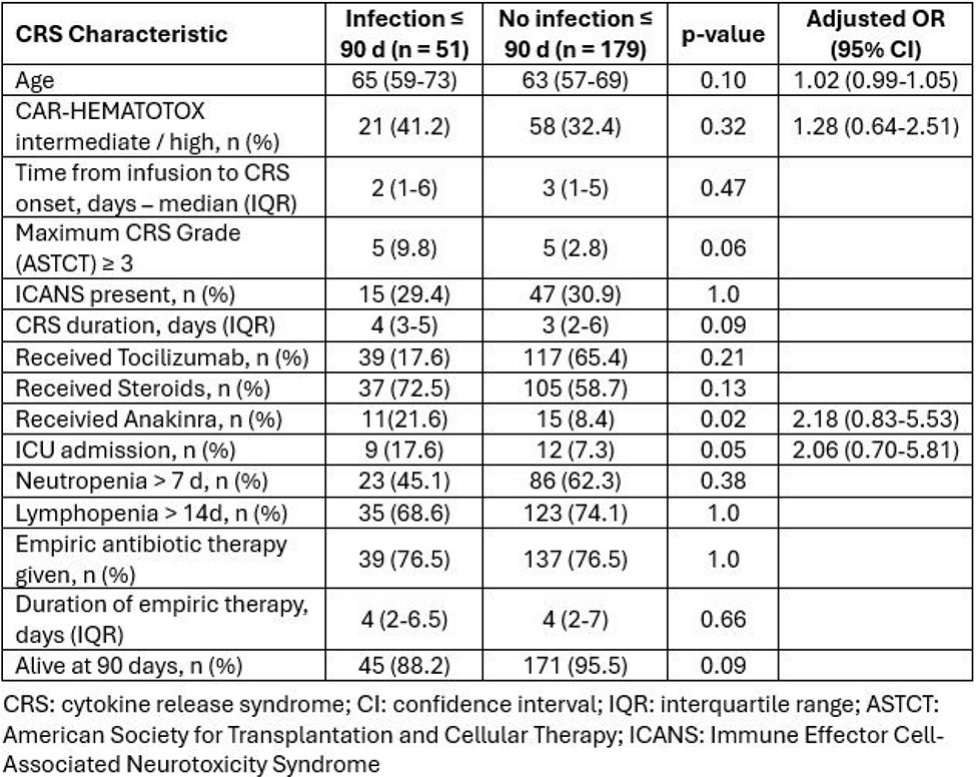
Cytokine Release Syndrome Severity, Management, and Early Outcomes by 90-Day Infection Status

**Methods:**

CAR-T recipients who developed CRS at our institution from 1 Jan 2018 to 3 June 2024 were retrospectively reviewed. Variables were abstracted into REDCap. CRS was graded with American Society for Transplantation and Cellular Therapy (ASTCT) criteria. All infections occuring after CRS onset to day 90 were adjudicated. Continuous data are reported as medians with interquartile ranges (IQR); categorical data as counts and percentages. Table 2 comparisons used Mann-Whitney U for continuous and χ² or Fisher exact tests for categorical variables (two sided, P < 0.05). Multivariable logistic regression was used to identify independent predictors of infection.

**Table 3:**
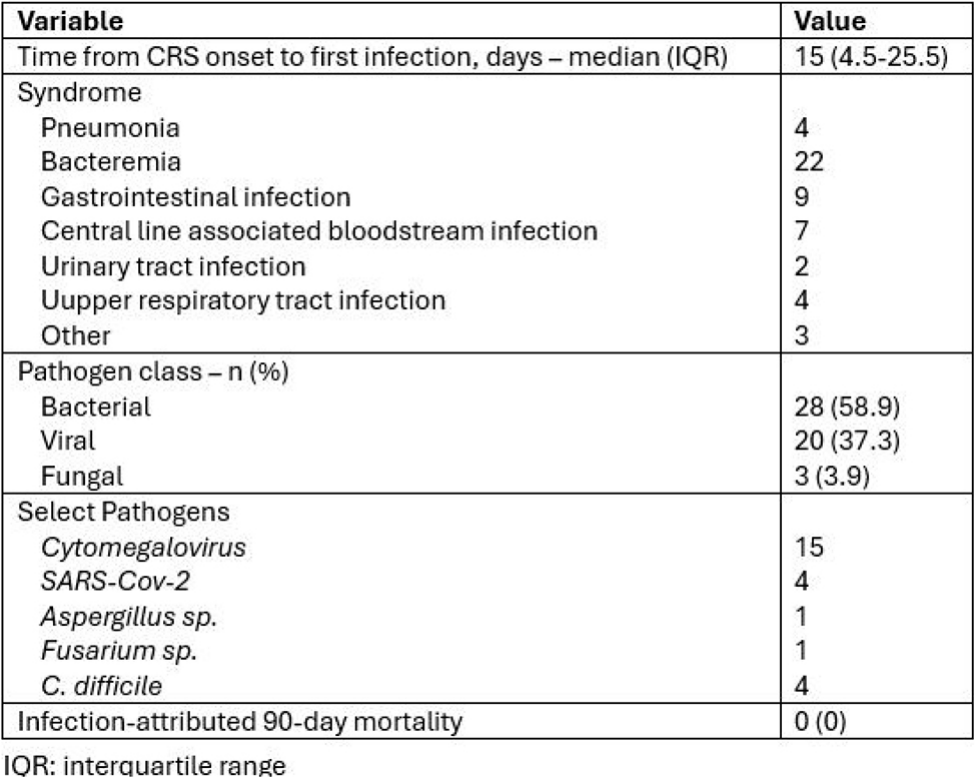
Infectious Syndromes, Pathogens, and Timing of First Infection Following CRS Onset

**Results:**

Among 230 CRS cases, median age was 64 yr (IQR 57–70) and 59% were male. Non-Hodgkin lymphoma accounted for 62% and multiple myeloma for 38%. Median prior therapy lines were 3. CRS was grade 1–2 in 97%. CAR-HEMATOTOX was low in 156 (68%) (Table 1). Within 90 days, 51 patients (22%) developed infection; median onset was 15 days (IQR 5–26). Infected patients more often received anakinra (22% vs 8%, p = 0.02) and required ICU care (18% vs 7%, p = 0.05); grade ≥3 CRS trended higher (10% vs 3%, p = 0.06). In multivariable analysis adjusting for age, CAR-HEMATOTOX, ICU admission, and anakinra, both ICU admission (aOR 2.06; 95% CI 0.70–5.81; p = 0.18) and anakinra use (aOR 2.18; 95% CI 0.83–5.53; p = 0.10) were associated with higher infection odds, though not statistically significant. 90-day survival was 88% with infection versus 96% without (p = 0.09) (Table 2). 51 infectious episodes were documented: bacteremia 22; gastrointestinal 9; central line–associated bloodstream infection 7; other sites 22. Bacteria caused 59%, viruses 37% (15 *CMV*, 4 *SARS-CoV-2*), and fungi 4%. No infection-related deaths occurred (Table 3).

**Conclusion:**

Infection was common after CRS, but did not alter early survival. Anakinra exposure and ICU admission were associated with a higher likelihood of infection, though not statistically significant. Targeted surveillance should focus on patients requiring intensified CRS management.

**Disclosures:**

Yi Lin, M.D., BMS: Board Member|Caribou: Advisor/Consultant|Genentech: Board Member|Jassen: Board Member|Jassen: Steering Committee|Kite/Gilead: Steering Committee|Legend: Board Member|NexImmune: Advisor/Consultant|NexT Therapeutics: Board Member|Pfizer: Board Member|Regeneron: Board Member|Sanofi: Board Member|Tessera: Board Member Paschalis Vergidis, MD, MSc, Current Fungal Infection Reports: Honoraria|F2G: Grant/Research Support|Merck Manuals: Honoraria|Mundipharma: Grant/Research Support|Scynexis: Advisor/Consultant|Scynexis: Grant/Research Support

